# Maternal stress and adolescent brain structure and function

**DOI:** 10.1002/brb3.1311

**Published:** 2019-05-14

**Authors:** Claire E. Niehaus, Tara M. Chaplin, Stefanie F. Gonçalves, Robin Semelsberger, James C. Thompson

**Affiliations:** ^1^ Department of Psychology George Mason University Fairfax VA

**Keywords:** adolescence, fMRI, mPFC, parent cortisol, parent stress, sMRI

## Abstract

**Introduction:**

Adolescence is a time of heightened sensitivity in biological stress systems and the emergence of stress‐related psychopathology. Thus, understanding environmental factors in adolescence that might be associated with adolescents'’ stress systems is important. Maternal stress levels may be involved. However, the relationship between maternal stress and the adolescent brain is unknown.

**Method:**

The present study examined the association between mothers' self‐reported stress levels and mothers' cortisol stress reactivity and their early adolescents' brain structure and functional activation to stressful negative emotional images. Participants included 66 mothers and their 12‐ to 14‐year old adolescents. Mother's perceived stress and salivary cortisol reactivity to a stressful task were collected. Then, adolescents' brain structure and function were assessed in a magnetic resonance imaging session.

**Results:**

Functional whole‐brain analyses revealed that mothers' higher reported perceived stress, but not cortisol reactivity, predicted adolescents' higher responses in the medial prefrontal cortex (mPFC) to stressful negative emotional stimuli. There were no statistically significant associations for structural analyses.

**Conclusions:**

Given the finding of maternal stress reactivity related to adolescent mPFC function—an integral structure related to stress responses—parent stress may play a role in the development of neural stress systems in adolescence, with potential implications for development of psychopathology.

## 1 INTRODUCTION

Adolescence is a time characterized by heightened stress system response thought to contribute to the spikes in psychopathology in adolescence (Grant, Compas, Thurm, McMahon, & Gipson, 2004; Gunnar, Wewerka, Frenn, Long, & Griggs, 2009). Research has tried to determine what predicts individual differences in adolescent stress system changes (Eiland & Romeo, 2013). One thing that could contribute is a mother's own stress levels. Research suggests that parents can affect youth's developing stress systems (Gunnar, 2017). Although there is some research finding that maternal stress (as assessed by mothers‐reported stress and cortisol responses) is correlated with child behavioral and peripheral stress system reactivity (e.g., cortisol stress reactivity) mostly in infancy, it is unclear if maternal stress responses are associated with adolescent neural stress system structure and function (Gutteling, de Weerth, & Buitelaar, 2005; O'Connor et al., 2005; Talge, Neal, Glover, & ES, TRPSN, 2007).

The present study examined associations between maternal perceived stress levels and cortisol stress reactivity and their adolescents' neural stress system structure and function using magnetic resonance imaging (MRI). Neural stress systems are rapidly developing during adolescence and are more sensitive to insult in adolescence than in childhood and adulthood (Lupien, McEwen, Gunnar, & Heim, 2009), making this a time in which maternal stress levels may particularly affect and shape youth's stress systems.

### 1.1 Adolescent neural stress system

There are three main brain regions that are involved in stress reactivity: the hippocampus, the prefrontal cortex (PFC), and the amygdala. The hippocampus is involved in shutting down the stress response and fear‐related learning, the PFC is involved in regulating the stress response and cognitive appraisal of the stressor, and the amygdala is involved in activating the stress response (Etkin, Enger, & Kalisch, 2011; Lupien et al., 2009). Two of these regions—the PFC and amygdala—are developing rapidly in adolescence (Gunnar & Herrera, 2013; Gunnar et al., 2009). Also, during adolescence, these regions may be particularly sensitive to environmental inputs, including maternal stress levels. For example, environmental factors during adolescence result in greater glucocorticoid exposure on the brain, affecting structure and function of stress regions, than similar environmental exposure later in life (Avital & Richter‐Levin, 2005).

### 1.2 Theory: Maternal stress and adolescent stress system function

It is important to examine whether maternal stress is associated with (and perhaps affects) child neural stress system structure and function. Social Learning Theory (Bandura, 1978) posits that children learn responses and behaviors that are modeled for them, in this case by their mother. Consistent with this, mothers who are highly stressed may model heightened stress responses to their adolescents who may then evidence structural and functional changes in their neural stress system, perhaps particularly in the amygdala and prefrontal cortex. Also, given that harsh parenting has been shown to affect youth stress responses (Jaffee et al., 2015), mothers with higher stress responses may parent more harshly and consequently impact their child's stress system (e.g., Martorell & Bugental, 2006). In any situation, it is important to first understand if maternal stress is correlated with adolescent neural stress system structure and function. If the two are related, interventions can target maternal stress levels and responses to modify adolescents' stress‐related brain structure and function and perhaps reduce the development of stress‐related psychopathology.

### 1.3 Previous research on maternal stress reactivity and child stress reactivity

Empirical studies support these theoretical claims of associations between maternal stress levels and the child stress system. For instance, several studies found associations between prenatal reported and hormonal maternal stress levels and later infant and child cortisol levels, providing evidence that maternal stress impacts the development of offspring stress systems in utero (Gutteling et al., 2005; Talge et al., 2007). Studies have found that maternal and infant salivary cortisol responses to a stressor are highly correlated and that heightened reported prenatal stress predicts child heightened cortisol response on the first day of preschool (Gutteling et al., 2005; Talge et al., 2007). Studies have also found that postpartum self‐reported heightened perceived stress levels predict later heightened cortisol response to stress of their preschool‐aged or preadolescent‐aged child (Gutteling et al., 2005; O'Connor et al., 2005). In older children and early adolescents (9–11 year olds), Banez and Compas have also found that mother‐reported daily hassles predict child reported daily hassles (1990). Associations between maternal stress responses and child stress systems in the postnatal period, during preschool, and during early adolescence suggest that the relationship between mother and child stress responses continues after birth and into early adolescence. However, little is known about the connection between maternal stress and adolescent neural stress system structure and function.

Some studies, while they do not examine maternal stress, support that life stressors in childhood or early adolescence (which may include maternal stress) are related to structure of the PFC and amygdala. For instance, preadolescents (8–11 year olds) exposed to high levels of life stressors (e.g., being institutionally raised, having mothers with high levels of depression), exhibit larger amygdala volumes and higher levels of anxiety (Lupien et al., 2011; Tottenham et al., 2010). Moreover, some evidence suggests that prefrontal cortex gray matter volume is attenuated in adolescents who experience life stressors (e.g., abuse) *during* adolescence (at ages 14–16) (Anderson et al., 2008). While the hippocampus is a key stress region in the brain, evidence suggests that early (e.g., ages 3–5), but not adolescent life stress is associated with reduced hippocampal volume (Anderson et al., 2008; Lupien et al., 2009), supporting a sensitive period hypothesis whereby areas developing at the time (e.g., amygdala and prefrontal cortex) are most susceptible to the effects of stress. Additionally, one study begins to explore the impact of maternal feelings of anxiety and possibly stress on offspring neural functioning. This study found that maternal high reported feelings of anxiety in the second trimester of pregnancy were associated with greater electroencephalogram right prefrontal activation while infants (20 weeks old) were in a quiet alert state (Field et al., 2003). Overall, stressors may be associated with changes in the adolescent brain and environmental factors, including maternal stress, could impact the structure and function of the developing adolescent brain. This may be particularly true in the amygdala and PFC. No research has examined links between maternal stress and child or adolescent neural stress system structure or function.

### 1.4 Current study

To address the questions of whether maternal stress is associated with adolescent neural stress system structure and function, we examined the impact of two forms of maternal stress responses (maternal‐reported perceived stress levels and maternal cortisol stress reactivity to a stressful Parent‐Adolescent Interaction Task (PAIT)) on adolescent brain structure and function using structural and functional magnetic resonance imaging (sMRI and fMRI). We chose perceived stress because maternal perceptions of their stress levels have been uniquely associated with child's biological stress system (e.g., cortisol) (Essex, Klein, Cho, & Kalin, 2002). And we chose cortisol reactivity in parent–child interactions because parent physiological stress has also been associated with child cortisol responses and internalizing symptoms, possibly through effects on the child's brain (Johnson & Gans, 2016; Papp, Pendry, & Adam, 2009). Adolescent brain function was examined during a stressful negative emotional images task. This was chosen to probe youth brain function during negative emotion processing, which might be particularly affected by maternal stress levels because maternal stress is associated with the development of child emotion processing systems and adolescence is a time in which emotion systems are sensitive to environmental effects (Casey, Jones, & Hare, 2008; O'Connor, Heron, Golding, & Glover, 2003). Also, function in emotion processing tasks has been affected by other forms of life stress in prior studies (Suzuki et al., 2014). We hypothesized that higher maternal‐reported stress levels and cortisol stress reactivity would predict adolescents' amygdala and prefrontal cortex structure and function. These areas have been implicated in stress responses and previous research of the impact of maternal stress on offspring in human infants and animals. However, given a dearth of research on how maternal stress may impact human adolescent brain structure and function, we did not hypothesize a direction for effects. In addition, we used a whole‐brain analysis approach so that we could detect if there were effects of maternal stress on structure and function in other brain regions outside of amygdala and PFC.

## 2 METHOD

### 2.1 Participants

Participants were 66 12‐ to14‐year olds (34 boys; Mean age = 12.59, *SD* = 0.70) and their mothers (95% biological mothers) were recruited from a larger study of emotion and parent–adolescent interactions. The majority of participants was European American (77.3%, 6.1% Asian American, 1.5% Hawaiian or Pacific Islander, 1.5% African American, 13.6% mixed/other; 9.1% Hispanic/Latino, 90.9% not Hispanic/Latino) and most had family household annual incomes above $100,000 (77.3%; 6.1% between $75,000–100,000; 10.6% between $60,000–74,999; 1.5% between $50,000–59,999; 3% <$50,000, 1.5% not reported). In the larger study, families were recruited through mailings to households in a suburban area in the mid‐Atlantic United States. Inclusion criteria for the larger study were that adolescents had an IQ ≥80 (on Wechsler Abbreviated Scale of Intelligence; Wechsler, 1999) and adequate English proficiency for the mother and adolescent to complete questionnaires. Exclusion criteria were prenatal substance exposure, low‐functioning Autism, or psychotic disorder for adolescents. Families were then selected from this study to participate in an additional fMRI session based on interest and fMRI eligibility (no metal braces, not pregnant, no congenital birth defect, or history of traumatic brain injury, and no current psychiatric medication use). The first 81 adolescents who were interested and eligible were invited to the fMRI session.

The larger study included 197 adolescents and their mothers. Of those 197 families, 81 participated in the additional fMRI session. Eighty‐one participants were chosen based on a power analysis to achieve 80% power to detect effects of parent stress on adolescent neural stress systems (G*Power; Faul, Erdfelder, Lang, & Buchner, 2007). The power analysis was based on previous studies which found a medium effect of adolescent brain responses in stress or emotion‐related regions associated with adolescent outcomes and accounting for having to exclude an expected 25% of subjects due to head motion or exiting the scanner (Hall et al., 2014). Of the 81 participants, nine were excluded because of inability to complete the scan and six were excluded due to excessive head motion, leaving a final sample of 66 adolescents that did not differ significantly from the larger sample regarding demographics (e.g., race, gender, and income), reported stress levels, or cortisol stress reactivity.

### 2.2 Procedures

**Figure 1:**
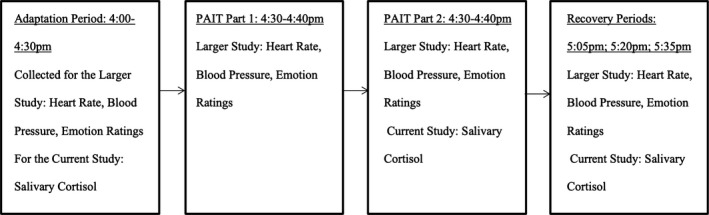
Mother's lab session measures and timeline. PAIT, Parent‐Adolescent Interaction Task

Families attended three sessions. The first session was a baseline questionnaire/ interview session. At this session, information regarding mother and adolescent psychopathology and substance use symptoms were collected for the larger study and mother‐reported demographics, stress levels, negative life events, and depressive symptoms were collected for the current study. The next session was a parent–adolescent interaction laboratory session, about 1–2 weeks later. In this session, mothers and their adolescents participated in an interaction task for the larger study and gave salivary cortisol samples before, immediately after, and during recovery from the Parent‐Adolescent Interaction Task (PAIT) for the current study. Timeline and Measures for the laboratory session are described in Figure 1. Lastly, eligible and interested families from the baseline session were recruited for the additional MRI session 1–2 weeks after the interaction session during which structural brain images and a reward and emotion task data were collected for the larger study and the structural images and emotion task data were used for the current study. For four adolescents, fMRI sessions were delayed 4–6 months due to adolescents having orthodontic braces. Those four adolescents did not differ significantly from the rest of the group on measures of mother cortisol stress reactivity, mother‐reported stress or depression levels, demographics, or family negative life events.

### 2.3 PAIT session

The parent–adolescent interaction lab session portion of the study was scheduled at 4 p.m. where possible in order to account for changes in cortisol levels throughout the day. About 89.2% of families were scheduled within one hour of 4 p.m. and the remaining 10.8% were scheduled within 1 hr and 45 min of 4 p.m. Number of minutes from 4 p.m. was unrelated to cortisol values in this study (*p* > 0.05). The PAIT task included two 10‐min stressful discussions: a conflict topic discussion and substance use discussion. For the conflict task, before the start of the PAIT task, the mother and adolescent separately completed the Issues Checklist (IC; Prinz, Foster, Kent, & O'Leary, 1979), a checklist of common parent–adolescent conflict topics that has been used in prior research with adolescents (e.g., Sheeber, Hops, Alpert, Davis, & Andrews, 1997). The mother–adolescent dyad indicated which conflict topics they had discussed in the past month and the level of anger they experienced while discussing those ranging from 1 (calm) to 5 (angry). The mutually highest rated conflict topic was selected as the discussion topic for the PAIT task. If the two differed in their highest rated topic, the mother's highest rated topic was selected. Preceding the PAIT task there was a 20‐min adaptation period during which the mother and adolescent went into separate rooms and listened to guided relaxation tapes for 5 min then sat quietly in a dim room without distractions for 15 min. During this adaptation period and at least 30 min prior to the adaptation, the mother and adolescent were asked to abstain from eating or drinking given the effect of food and drink intake on cortisol levels.

After the 20‐min adaptation period, baseline maternal salivary cortisol samples were collected. Next, the mother was brought into the room with the adolescent and seated next to him or her. Then the two discussed the selected conflict topic for 10 min. The dyad was asked to try to come to a resolution for the issue and was instructed to continue talking for a full 10 min and “discuss the issue as if you were at home.” Then mothers and adolescents completed a second task, a parent–adolescent discussion about substance use for 10 min where parents and their adolescent were asked to “discuss the topic of using alcohol, tobacco, marijuana, or any other drug for 10 min.” Again, if the family finished early, they were asked to keep talking. Maternal cortisol was collected immediately following this task and for two 15‐min intervals after the task while the mother was recovering from the PAIT task. That is, cortisol was collected at six times at the PAIT session: once when arriving at the lab, once immediately before the conflict task (after adaptation period), once immediately following the tasks, and three times during the recovery period. We also collected mother's self‐report of her in‐the‐moment stress or anxiety levels on a 1 (“not at all”) to 10 (“more than ever”) immediately before and after the task to assess whether the task evoked stress.

### 2.4 fMRI session

Adolescents completed a structural scan, resting state scan, and an emotional image scan. The stressful negative emotional image scan was a rapid event‐related design that included 27 negative, 27 neutral, and 27 positive images taken from the International Affective Picture System (IAPS; Lang, Bradley, & Cuthbert, 2008). The IAPS is a standardized method normed for valence and arousal levels designed to evoke emotional responses. IAPS pictures have been found to elicit cortico‐limbic activation in adolescents (McRae et al., 2012). Based on a previous study with adolescents (McRae et al., 2012), we selected IAPS pictures that were developmentally appropriate. Negative, neutral, and positive images were matched on subject type, color, and luminescence. Images were presented in pseudo‐randomized order, with trial order and timing determined by Optseq2 (Dale, 1999). Trials were presented across three runs each lasting about 6.5 min with a balanced number of trial types per run. Each trial consisted of viewing a picture (4s), youth rating their intensity of negative emotion (2s) and positive emotion (2s) on a scale from 1 to 4 using a button box, and an inter‐trial interval period (viewing crosshairs) jittered between 2s and 12s.

### 2.5 MRI image acquisition

Functional and structural images were acquired on a Siemens 3T Allegra MR scanner. Functional images of the blood oxygen‐level dependent (BOLD) response during the emotion task were collected using T2*weighted gradient‐echo echo planar images (EPI) [TR/TE: 2,250/30ms; flip = 70°; field of view (FOV): 192mm; matrix size: 64 × 64; 40 axial 3 mm thick/1mm gap slices]. For structural imaging, a whole‐head T1‐weighted magnetization‐prepared rapid‐acquisition gradient echo (MPRAGE) anatomical image was acquired (TR/TE = 2,300/3ms; FOV = 260 mm; matrix size = 256 × 256; 160 1mm thick slices).

### 2.6 Measures

#### 2.6.1 Maternal‐reported stress levels

At the baseline questionnaire/interview session, mothers reported on their perceived stress levels using the Perceived Stress Scale (PSS; Cohen, Kamarck, & Mermelstein, 1994). The PSS is a 14‐item Likert‐type questionnaire with statements about stress levels in the past month (e.g., “In the past month, how often have you felt difficulties were piling up so high you could not overcome them?”) and responses ranging from 0 (Never) to 4 (Very Often). The PSS has demonstrated good reliability and validity (Cohen et al., 1994). In the current study, the PSS also demonstrated good reliability (*α* = 0.83).

#### 2.6.2 Family stressors

At the baseline questionnaire/interview session, adolescents reported on family stressors using the Negative Life Events Inventory (NLEI; Wills, Sandy, Yaeger, Cleary, & Shinar, 2001). The NLEI is a 20‐item checklist of negative events in the family over the past year (e.g., “My father/mother lost his/her job”). Since one possible reason maternal stress may impact the adolescent brain in stress‐related regions is that both the mother and child have shared environmental stressors, we explored the effect of life events on the adolescent brain in our analyses as a potential covariate. The NLEI has demonstrated good validity in previous studies (Wills et al., 2001) although reliability is not often measured on the NLEI since it is an event checklist rather than questions intended to hang together.

#### 2.6.3 Maternal depression

At the baseline questionnaire/interview session, mothers also reported on their levels of depression using the Center for Epidemiological Studies Depression Scale (CES‐D; Radloff, 1977). Since previous research has found maternal depression is associated with child–brain responses (e.g., Dawson et al., 2003) and depression and stress are related (see Hammen, 2005 for a review of this relationship), we examined maternal depressive levels as a potential covariate in our analyses. This allowed us to see if maternal depression could explain the relationship between maternal stress and the adolescent brain. The CES‐D is a widely used measure of depressive symptoms with adults in various contexts and has demonstrated good reliability and validity in previous studies (Radloff, 1977). In the present study, the CES‐D also demonstrated good reliability (*α* = 0.82).

#### 2.6.4 Cortisol stress reactivity

Mother saliva was collected using a cotton swab that mothers placed between their tongue and their cheek until adequately saturated (about 2 min). Consistent with other studies (e.g., Buss, Davidson, Kalin, & Goldsmith, 2004), saliva samples were assayed in duplicate using standard radioimmunoassay kits with no modifications (intra‐assay coefficients of variation from 3.0% to 5.1%). Cortisol response values were calculated using a maximum minus minimum value of cortisol over the six measurements, as suggested to capture cortisol reactivity in repeated measures by Miller and colleagues (2018). For descriptive statistics on cortisol at each timepoint and self‐report measures, see Table 1. Notably, cortisol values were decreasing across our measurements, consistent with studies that show that cortisol levels typically decrease in the evening (Chaplin et al., 2012; Horrocks et al., 1990). However, individual differences in the extent to which cortisol decreased or did not decrease from pre‐ to post‐PAIT may be associated with child outcomes as has been shown in prior research (citation masked for review). However, maternal report of anxiety or stress levels (on a 1 to 10 scale) significantly increased from immediately before to immediately after the tasks in paired *t*‐test analyses (*t* = −4.40, *p* < 0.001) suggesting the tasks did evoke perceived stress.

**Table 1:** Descriptive statistics

	Mean (*SD*)
Parent cortisol (ug/dl)	
Baseline	0.21 (0.16)
After PAIT Task	0.20 (0.14)
25 min after PAIT Task	0.19 (0.15)
40 min after PAIT Task	0.16 (0.13)
55 min after PAIT Task	0.15 (0.13)
70 min after PAIT Task	0.14 (0.12)
Perceived Stress Scale (PSS)	18.92 (6.96)
*Cortisol Maximum‐Minimum*	*1.18 (0.75)*

Abbreviations: *SD*, standard deviation; Ug/dl, microgram/deciliter; PAIT, Parent‐Adolescent Interaction Task.

### 2.7 Analysis plan

#### 2.7.1 Missing data

Maternal‐reported stress levels on the PSS, the CES‐D, NLEI, and adolescent brain data did not have any missing data. However, we had five missing cortisol response values. Although our diagnostic tests revealed that the missing data were missing at random, we wanted to retain this substantial portion of our data and thus used multiple imputation in SPSS as a strategy for estimating the missing cortisol values. Multiple imputation creates multiple datasets (*n* = 10 datasets) replacing missing values with plausible values from the observed data then combining these values to produce estimates of the missing values (Little & Rubin, 2002). These different estimations were then pooled to replace the missing cortisol values. Structural and functional MRI analyses were run with the full dataset including these imputed values for cortisol.

#### 2.7.2 Covariates

We considered 5five covariates for our analyses: maternal depression, maternal negative life events, child age, gender, and length of time between the PAIT and fMRI session (since 4 adolescents were scanned 6 months after the PAIT session). It is possible that maternal depression levels and negative life events that may be shared by parent and child could account for maternal stress to child brain associations. To address this possibility, we ran structural and functional whole‐brain models with maternal‐reported depression and negative life events as predictors to determine if these factors were related to the child's brain and possibly would explain maternal stress to child brain relationships. The whole‐brain functional and structural analyses for these two variables did not reveal any significant clusters at *z* > 2.6 (*p* < 0.01) threshold for functional analyses and a Monte‐Carlo correction for multiple comparisons with 10,000 iterations for structural analyses. Thus, our final results do not covary these maternal depression or negative life event variables. However, we did add length of time between the PAIT session and fMRI session as a covariate to control for possible time effects in our analyses. Additionally, in the structural analyses we added child age and gender as covariates due to well‐documented differences in cortical volume by gender and age (Luders et al., 2006; Wierenga, Langen, Oranje, & Durston, 2014).

### 2.8 Statistical methods

#### 2.8.1 fMRI preprocessing and preliminary analyses

fMRI data were analyzed using FMRIB's Software Library version 5.0 (FSL; Jenkinson, Beckmann, Behrens, Woolrich, & Smith, 2012). Data were motion corrected, B0 unwarped, and slice‐timing corrected. Additionally, a 1/96 high pass temporal filter was applied to the data to remove frequency drifts. Then data were co‐registered to each subjects MPRAGE anatomical image then the Montreal Neurological Institute (MNI) template. Subjects with motion outliers (motion exceeding 3 mm for one TR or exceeding 1.5 mm for ≥80% of the TRs) were excluded from analyses. First level analyses were conducted using FMRIB's Improved Linear Model (FILM). In first level analyses, regressors were added for onset and duration of events and convolved with a double‐gamma hemodynamic response function. Nuisance regressors for motion correction parameters were added and remaining runs with motion greater than 1.5 mm (but less than 3 mm) for one TR were run through FSL's motion outlier function. Data were also prewhitened. Next, multiple regressions estimated the effect of the task. BOLD responses to negative and neutral images were used to create our contrast of interest (negative > neutral). This contrast reflects BOLD activation to negative emotional (and likely stressful) stimuli, controlling for general activation to visual stimuli.

#### 2.8.2 Functional whole‐brain analyses

Given that the effect of maternal stress on adolescent brain responses has not been examined before, we decided to conduct whole‐brain analyses to test our hypothesis that maternal stress would impact adolescent brain function in response to stressful negative emotional images rather than targeted region of interest (ROI) analyses. Our analyses focused on the association between adolescent whole‐brain response to the negative > neutral condition of our emotion task and maternal stress (reported stress levels and cortisol stress reactivity), covarying length of time between the PAIT and fMRI session. Whole‐brain analyses were conducted using FSL's FLAME mixed effects model and due to the possibility of inflated *p* values with multiple comparisons were corrected for multiple comparisons using a voxel‐based threshold of *z* > 2.6 and cluster‐based correction of *p* < 0.05.

#### 2.8.3 Structural whole‐brain analyses

For our structural analyses, data were preprocessed by resampling data to an average participant using the qcache command and surface smoothed using a 20 mm full‐width half maximum (FWHM) Gaussian kernel. Next, data were visually inspected for accuracy of segmentation by a researcher trained in structural MRI analyses and any errors in segmentation were manually fixed and rerun through qcache using the recon‐2 command. Then, this cleaned data were analyzed for differences in cortical thickness related to maternal‐reported stress levels and maternal cortisol stress reactivity, covarying child age, gender, and whole‐brain volume to control for changes in brain volume across development and by gender, at the group level using Freesurfer's qdec (http://surfer.nmr.mgh.harvard.edu/fswiki/FsTutorial/QdecGroupAnalysis). Qdec uses the general linear model (GLM) to model thickness at each surface vertex as a linear combination of effects related to variables of interest. Next, to address the possibility of inflated *p* values with multiple comparisons, data were corrected for multiple comparisons using Monte‐Carlo permutations with 10,000 iterations and mapped onto the Desikan–Killiany atlas.

## 3 RESULTS

### 3.1 Functional activation results

**Figure 2:**
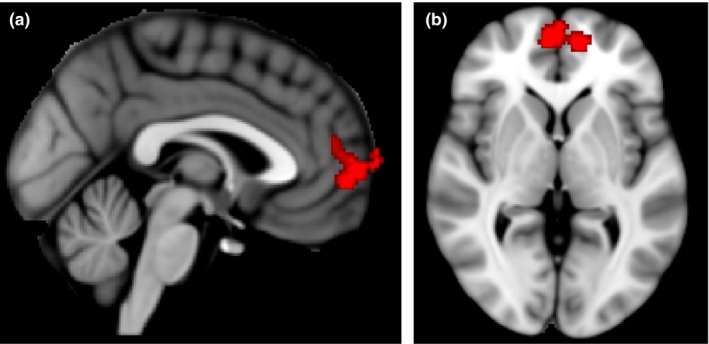
Sagittal (left) and axial (right) view of BOLD activation in adolescent mPFC during negative > neutral contrast correlated with parent reported stress levels (*z* = 3.34, *p*<0.05, MNI coordinates −18, 52, 10)

The whole‐brain functional activation analysis examined mother‐reported stress levels predicting adolescent whole‐brain BOLD response to the negative > neutral contrast, with length of time between the PAIT and fMRI session included as a covariate and a *z* > 2.6 (*p* < 0.01) threshold was used. Results revealed that higher mother‐reported stress levels were associated with higher adolescent BOLD response in the bilateral medial prefrontal cortex (mPFC) in response to the negative > neutral contrast with *z* > 2.6 threshold (*z* = 3.34, *p* = 0.0008; MNI coordinates, −18, 52, 10), controlling for length of time between the PAIT and fMRI session. See Figure 2 for images of this cluster. Length of time between PAIT and fMRI session, did not significantly predict the mPFC cluster at the *z* > 2.6 threshold.

Maternal cortisol stress reactivity to the PAIT task was not related to adolescent BOLD responses to negative > neutral contrasts.

### 3.2 Structural results

The whole‐brain structural analyses examined mother‐reported stress then maternal cortisol reactivity levels predicting adolescent cortical thickness with length of time between the PAIT and fMRI session and child age, gender, and whole‐brain volume included as a covariates. Results of whole‐brain structural analyses focusing on adolescent cortical thickness related to mother‐reported stress levels and cortisol stress reactivity with our covariates revealed no association between mother stress response variables and adolescent cortical thickness after Monte‐Carlo corrections for multiple comparisons with 10,000 iterations.

## 4 DISCUSSION

Our results revealed that higher maternal‐reported stress levels were significantly associated with higher adolescent BOLD response in the mPFC to stressful negative emotional stimuli. We did not find associations between maternal cortisol stress reactivity and adolescent neural responses to negative emotional stimuli, in contrast to our hypotheses. We also did not find associations between maternal‐reported stress levels or maternal cortisol stress reactivity and adolescent stress system brain structure. The finding that maternal stress levels were associated with altered mPFC function in adolescents which is significant in that altered mPFC function may indicate important neural changes and risk for negative outcomes in youth.

It is interesting that we found higher maternal‐reported stress levels associated with *higher* mPFC activation in adolescents. Given that the mPFC is sometimes conceptualized as having a stress regulatory role, one might expect to see reduced mPFC activation in higher risk youth who have been exposed to maternal stress. There are at least two possibilities for why we would see higher mPFC activation. First, if the mPFC *is* taking a regulatory role, perhaps children of highly stressed mothers overregulate or over‐engage the mPFC to regulate in the face of stressful negative emotional stimuli because they have been chronically exposed to highly stressed mothers. In this case, this overuse of mPFC in the face of stress may in time exhaust regulatory resources and/or lead to changes in the mPFC that could put youth at risk for psychopathology. A second explanation follows from findings that heightened mPFC activation is associated with self‐referential processing and appraisal of stress and negative emotion (Etkin et al., 2011). Thus, adolescent heightened activation in mPFC during an emotionally evocative task may indicate heightened processing of and reactivity to stressful negative emotional stimuli in adolescents whose mothers report high stress. Notably, high mPFC activation to negative emotional stimuli has been associated with maladaptive thought processes like rumination and associated with depression, possibly due to the role of the mPFC in self‐referential focus and stress processing and reactivity (Berman et al., 2014; Etkin et al., 2011; Smith, Baxter, Thayer, & Lane, 2016; Wagner, Müller, Sommer, Klein, & Hajak, 2004). We also know that mothers with higher stressful life events have youth with higher risk for psychopathology and poor adjustment (Conger, Patterson, & Ge, 1995). The implication of our findings is that maternal stress may lead to risk for adolescent psychopathology through their potential effects on mPFC activation.

Mother‐reported stress levels may be associated with adolescents' higher mPFC activation to stressful negative emotional stimuli through a number of mechanisms. One possibility is that mothers may model high stress responses to their children who then learn to respond to stress with heightened arousal, leading to the need for either greater regulation from mPFC or leading to more self‐referential processing and engagement of mPFC. Second, perhaps mothers who are more stressed have themselves altered neural stress system function, including mPFC function, that they genetically passed down to their children. This could be explored in studies that use neuroimaging with parents and their children in one study. Thirdly, mothers with high stress responses may parent more harshly (see Dix, 1991), which may lead to more stressed offspring reflected in higher neural stress system function. Now that this study has established a link between maternal‐reported stress levels and adolescent mPFC reactivity, future research can examine parenting behavior as a possible mechanism for this association.

Notably, we did not find significant associations between maternal stress and adolescent amygdala activation or activation in other stress‐related regions to the stressful negative emotional stimuli. This is surprising because the amygdala is involved in the neural stress response and is developed (and vulnerable to environmental effects) during adolescence (Lupien et al., 2009). Given that the amygdala is more involved in supporting the initial emotional activation response rather than regulation or appraisal of the response, it may be that this initial heightening of the stress/emotional response to negative emotional stimuli is less impacted by maternal stress than is appraisal and regulation. Alternatively, effects of maternal stress on the adolescent amygdala structure and function may be smaller effects that we were underpowered to detect.

Notably, we did not find whole‐brain results for maternal cortisol stress reactivity associated with adolescent brain response to stressful negative emotional stimuli. In previous literature, cortisol and reported stress have been positively correlated, thus one might expect that both would be related to the adolescent's brain (Pruessner, Hellhammer, Pruessner, & Lupien, 2003). However, there are a couple of reasons that cortisol and reported stress results may differ. First, mother's subjective appraisals of their stress levels may have more of an impact on how they parent or model stress responses than their actual raw hormonal stress response. Secondly, measurement differences might also explain the difference in results. Cortisol was measured on one occasion in the lab and maternal perceived stress was reported on in daily life in the past month. Thus, a mother's perception of stress in daily life may have more wide‐reaching associations with adolescent neural stress system than one instance of hormonal reactivity. Lastly, although cortisol was collected at a time when cortisol levels are typically falling in the day, our cortisol values did not increase from the task unlike other studies that use a stress task such as the Trier Social Stress Task (TSST; Dickerson & Kemeny, 2004). Thus, perhaps our task did not evoke a robust enough cortisol response to detect effects and future studies might consider using a task that has a social evaluative component such as the TSST which is thought to contribute to robust cortisol responses to that task (Dickerson & Kemeny, 2004).

Lastly, our structural analyses did not reveal any effect of maternal stress on adolescent cortical thickness, contrary to expectations given the documented effect of other environmental factors on brain structure (e.g., Romeo & McEwen, 2006). One possibility for not finding structural effects of maternal stress on the brain is that our sample is early adolescents—right when stress regions like the PFC and amygdala are developing—thus it may take more time for maternal stress levels to affect the structure of these newly developing regions. Perhaps an effect of maternal stress on adolescent brain structure would not be seen until later adolescence. It would be interesting to explore this question in a sample of older adolescents. Lastly, it is possible that these hypotheses were not supported due to low power to detect effects with our sample size. Since whole‐brain analyses do not provide information on marginally significant or nonsignificant results, future studies should examine region of interest analyses which do provide this information to determine if limited power could play a role in unsupported hypotheses.

Overall, this is the first study to examine how maternal stress may impact offspring brain structure and function during a time of heightened risk for the development of psychopathology and changes to stress‐related brain regions: adolescence. Results from the present study suggest that mothers' higher perceived stress levels are associated with adolescent heightened response in the mPFC to negative emotional stimuli, a region in the stress system implicated in emotion regulation, stress processing, and self‐referential processing and that has also been implicated in rumination and psychopathology. Although examining the association between maternal stress and youth stress systems at a neural level is a strength of our study, there are limitations of our study as well. One limitation is that this study was cross‐sectional making it impossible to discern the direction of effects. For instance, adolescents with more activation in the mPFC may exhibit behaviors that heighten their mother's stress levels or highly stressed mothers may model high stress responses and lead to adolescents having an increased neural response to stressful stimuli.

It would also be important to have a longitudinal study of these relationships to test our hypothesis that adolescents with higher mPFC reactivity may be at heightened risk for depression and other internalizing disorders. Secondly, whole‐brain analyses are empirically driven and thus a priori region of interest analyses may have yielded different results. Lastly, our sample is largely a high socioeconomic status and white sample and thus exploring these relationships in a more diverse sample may be helpful for prevention efforts in communities that may experience higher levels of stress. Overall, our results suggest that maternal‐reported stress levels are associated with increased adolescent neural reactivity to stressful emotional stimuli in the mPFC. Consequently, interventions to reduce maternal stress levels may be a worthwhile pathway to reducing adolescent risk for developing maladaptive neural responses to negative emotional stimuli and possibly even psychopathology.

## CONFLICT OF INTEREST STATEMENT

The authors declare no conflicts of interest.

## Data Availability

For this study, we have a data sharing plan with our funding source. Because much of this dataset is sensitive in nature or unable to be reduced to an easily sharable format like excel (e.g., whole‐brain data), our protocol is to have people interested in the data to email us directly and describe the analyses they plan to conduct and we will send them the appropriate data.
